# Prediction and Experimental Characterization of nsSNPs Altering Human PDZ-Binding Motifs

**DOI:** 10.1371/journal.pone.0094507

**Published:** 2014-04-10

**Authors:** David Gfeller, Andreas Ernst, Nick Jarvik, Sachdev S. Sidhu, Gary D. Bader

**Affiliations:** 1 The Donnelly Centre, University of Toronto, Toronto, Ontario, Canada; 2 Swiss Institute of Bioinformatics, Quartier Sorge, Bâtiment Génopode, Lausanne, Switzerland; 3 Department of Molecular Genetics, University of Toronto, Toronto, Ontario, Canada; 4 Department of Computer Science, University of Toronto, Toronto, Ontario, Canada; Weizmann Institute of Science, Israel

## Abstract

Single nucleotide polymorphisms (SNPs) are a major contributor to genetic and phenotypic variation within populations. Non-synonymous SNPs (nsSNPs) modify the sequence of proteins and can affect their folding or binding properties. Experimental analysis of all nsSNPs is currently unfeasible and therefore computational predictions of the molecular effect of nsSNPs are helpful to guide experimental investigations. While some nsSNPs can be accurately characterized, for instance if they fall into strongly conserved or well annotated regions, the molecular consequences of many others are more challenging to predict. In particular, nsSNPs affecting less structured, and often less conserved regions, are difficult to characterize. Binding sites that mediate protein-protein or other protein interactions are an important class of functional sites on proteins and can be used to help interpret nsSNPs. Binding sites targeted by the PDZ modular peptide recognition domain have recently been characterized. Here we use this data to show that it is possible to computationally identify nsSNPs in PDZ binding motifs that modify or prevent binding to the proteins containing the motifs. We confirm these predictions by experimentally validating a selected subset with ELISA. Our work also highlights the importance of better characterizing linear motifs in proteins as many of these can be affected by genetic variations.

## Introduction

Genome sequencing projects have uncovered thousands of genetic variations in human populations [1]. Cancer genome projects and genome-wide association studies are increasingly finding those that correlate with disease susceptibility [2]–[5]. A prevalent mode of genetic variation is Single Nucleotide Polymorphism (SNP), where one DNA base pair is exchanged for another. SNPs falling in protein coding regions and modifying the encoded amino acid are called non-synonymous SNPs (nsSNPs). nsSNPs result mainly in one amino acid being replaced by a different type at a given position in the protein sequence. Alternatively stop codons can arise, thereby truncating the protein sequence before its normal C-terminus, or be removed, resulting in an abnormally long protein.

SNPs can be rapidly identified using microarray or DNA sequencing technology and the number of reported nsSNPs in human proteins exceeds 10^6^. It is therefore important to understand and predict the functional and molecular consequences of nsSNPs and several computational approaches have been developed to address this issue [6]–[13]. These algorithms typically use sequence conservation, domain annotation, structural environment, biochemical similarity between the wild type and the mutated residues, or manual annotations based on detailed biochemical studies to predict the functional impact of nsSNPs. However, despite the large panel of methods that have been proposed, the functional consequences of nsSNPs are still difficult to predict. This is especially true in regions displaying low conservation, no evident structure (e.g., disordered regions) or poor annotation, which represent a substantial portion of human protein sequences.

Binding sites that mediate protein-protein or other protein interactions are an important class of functional sites on proteins and can be used to help interpret nsSNPs [13]. For instance, an nsSNP can alter a binding site to prevent or cause a protein-protein interaction and this can modify the function of the altered protein. Here we focus on nsSNPs affecting short linear motifs binding to peptide recognition modules, such as SH3, PDZ or WW domains [14]–[16], as these binding sites play important roles in cell signaling pathways [17]. Peptide recognition modules are protein interaction domains that recognize small stretches of amino acids exposed on the surface of their interactors. Such amino acids are frequently found in disordered segments. For instance, PDZ domains bind mainly to the C-terminus of proteins, which often do not adopt well-defined conformations on the protein surface and therefore can be targeted without requiring structural remodeling [18]. Because of the small binding interface, a single residue modification in a motif can completely disrupt the interaction with the peptide recognition modules binding to it [19], [20]. Therefore linear motifs can be highly sensitive to nsSNPs and nsSNPs in these motifs have been linked to several diseases [21]–[24].

Linear motifs are not easy to detect in a statistically significant way because they are short and remain poorly annotated in the human genome. One standard approach to identify them is to use consensus motifs (e.g., the PxxPx[R/K] motif for SH3 domains) and large collections of such motifs are available in databases [15], [25]. Using consensus motifs, some attempts have been made to predict the effect of nsSNPs in linear motifs [15]. Although successful predictions can be achieved for nsSNPs falling on key residues that are required for the binding, such as the two prolines in canonical SH3 domain ligands, predicting the effect of nsSNPs on positions that display less specificity is difficult using this approach.

Recently, together with an increase in available experimental data for these domains (e.g. [19]), more elaborate computational models have been devised to better represent the amino acid preferences at each position along the peptides interacting with a domain [26]–[29]. For instance, Position Weight Matrices (PWMs) have been used to capture the detailed amino acid preferences at each position of the ligands binding to a given domain [30]. These models have been successfully applied to predict new proteins interacting with domains such as SH3, PDZ or kinases [26], [31], [32].

Here we show that these models of specificity are also useful to determine nsSNPs affecting PDZ-binding linear motifs in human proteins. We observed several cases of nsSNPs that fall into PDZ-binding motifs and where the mutated residue is expected to significantly disrupt the interaction. Experimental validations for three of the most promising cases were carried out with enzyme-linked immunosorbent assays (ELISAs) to assess binding activity. In all three cases, the WT peptides were found to interact with the predicted PDZ domain, while the nsSNPs significantly disrupted the interaction. In particular, we found that the PDZ domain of CYTIP (also known as CASP or PSCDB) can bind the C-terminal peptide of PCDHA1, a member of the cadherin family. The PDZ domain of CYTIP is required for its correct localization to the cell cortex [33]. Our results suggest that the PCDHA1 (ENSP00000367373 in Ensembl version 74, isoform 3 in UniProt Q9Y5I3-3) C-terminus binds a PDZ domain, such as the one in CYTIP, and that this interaction may be disrupted by a C-terminal nsSNP in PCDHA1.

## Materials and Methods

### nsSNP identification

All nsSNPs from dbSNP [34] found in coding regions were initially retrieved using the mapping at the protein level from RefSeq (version from Sep 2, 2013). To identify nsSNPs that affect the C-terminus of a protein, we isolated nsSNPs falling in the last seven amino acids, which is the typical maximum length expected for a canonical C-terminal PDZ domain binding site [19]. For proteins displaying multiple splice variants, the C-terminal part of each variant was considered and an nsSNP was defined as affecting the C-terminal sequence of a protein if it falls in the C-terminus of any splice variant.

To scan human proteins for the presence of a PDZ binding motif, we first retrieved all human proteins from RefSeq (21,653 in total). As PDZ domains occur primarily in cytoplasm, we filtered out extracellular and mitochondrial proteins using Gene Ontology categories [35]. Extracellular proteins were determined by considering the union of GO:0031012 (extracellular matrix) and GO:0005615 (extracellular space). As several plasma membrane proteins are found in these two sets, we did not filter out proteins annotated with GO:0044459 (plasma membrane part). The final set of extracellular proteins consisted of 1056 proteins. Mitochondrial proteins were determined as those annotated with GO:0044429 (mitochondrial part, 854 proteins in total).

In total our filtering procedure resulted in 19,751 proteins with 13,490 nsSNPs consisting of a single residue variation in a C-terminus and 26,864 stop codons resulting in a different C-terminus. When computing statistics regarding nsSNPs in C-terminal regions, the frequency of nsSNPs in C-termini was compared to the frequency of nsSNPs anywhere in proteins using the Binomial test, since multiple nsSNPs can affect the same residue.

### Computational proteome scanning

Positions Weight Matrices (PWMs) were used to predict PDZ-binding motifs containing C-termini. For each PDZ domain, PWMs were built from phage display data [19], by aligning the interacting peptides at their C-terminus and computing the frequency of each residue at each position. In total, 54 PDZ domains found in 34 different proteins with available experimental data were considered in this study. A random count proportional to the information content at each position in the alignments was used to account for under-sampled residues. PDZ domains are referred to by the name of the protein they are found in. In case of multiple PDZ domains on the same protein the ‘#’ symbol is used to number them.

**Figure 1:**
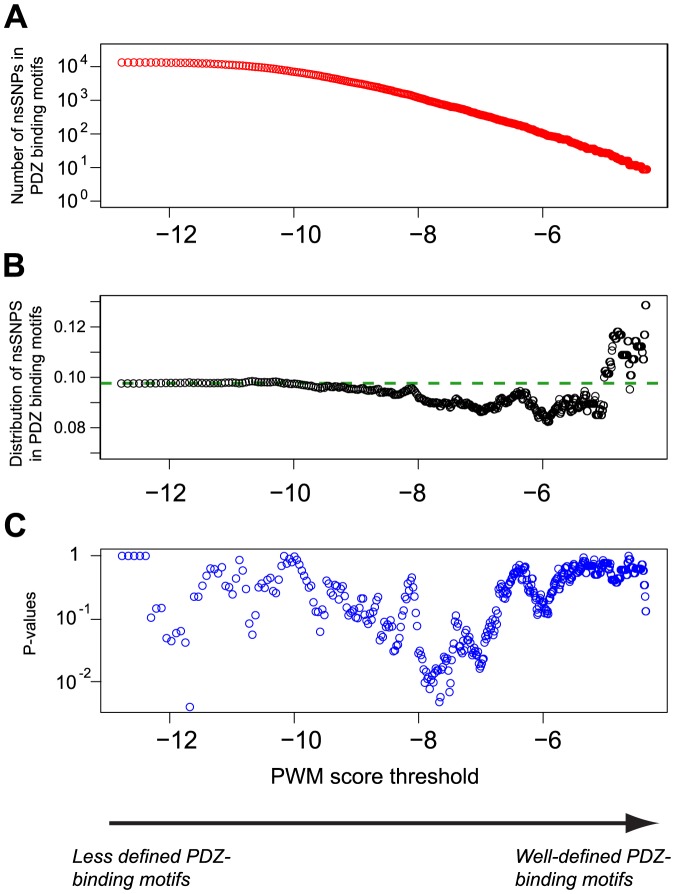
PDZ-binding linear motifs are affected by nsSNPs. A) Number of nsSNPs that are found in PDZ-binding motif containing C-termini for a wide range of thresholds on PWM scores to define PDZ-binding motifs. Higher threshold values (corresponding to more stringent definitions of PDZ-binding motifs) result in few C-termini being considered as containing a PDZ-binding motif, hence few nsSNPs falling in these motifs. B) Distribution of nsSNPs in PDZ-binding motifs, computed as the ration between the number nsSNPs shown in panel A and the total number of amino acids in PDZ-binding motif containing C-termini. For the highest thresholds (>−6), the number of nsSNPs falling in PDZ-binding motifs is not lower than expected. Then for thresholds between −6 and −9, it becomes slightly lower. For even lower thresholds, we tend to the expected distribution observed for all C-termini (9.9% of positions affected by nsSNPs, dashed line). C) The corresponding P-value assuming a uniform distribution of nsSNPs in all C-terminal segments (binomial test).

**Figure 2:**
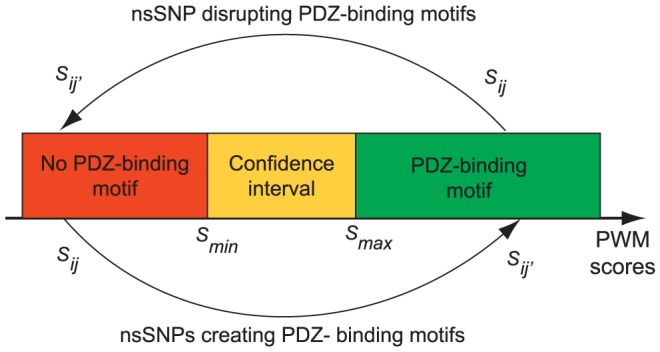
nsSNPs can disrupt a PDZ-binding motif, create a new one, or not have an effect. The two arrows illustrate our algorithm to predict the first two cases. A nsSNP is considered to disrupt a PDZ-binding motif if the wild-type sequence (*j*) has a score *S_ij_*>*S_max_* (green box), while the modified sequence (*j′*) has a score *S_ij′_*<*S_min_* (red box) with at least one PDZ domain (*i*) (upper arrow). Reversely, nsSNPs can create new PDZ binding motifs if *S_ij_*<*S_min_* and *S_ij′_* >*S_max_* (lower arrow). The confidence interval (yellow box) is used to ensure that the scores are different enough between the wild type and the mutant to predict nsSNPs effect on PDZ mediated interactions.

**Figure 3:**
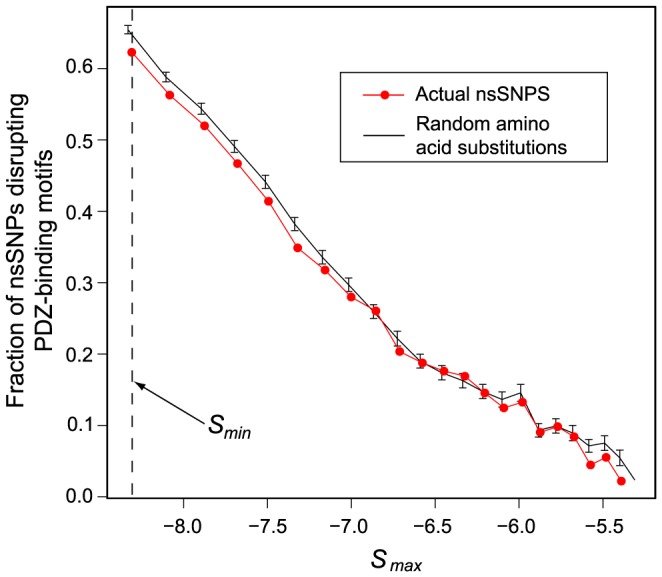
nsSNPs disrupting PDZ-binding motifs are only slightly under-represented in human proteins. The red curve shows the fraction of known nsSNPs predicted to disrupt PDZ-binding motifs for different values of the threshold *S_max_*. The black curve shows the same data for random amino acid substitutions on residues affected by nsSNPs in human C-termini (error bars show standard deviation for 1000 randomization of nsSNPs).

PWMs were used to score all human C-terminal sequences retrieved from the RefSeq database for each PDZ domain. The final score between a domain *i* and a protein *j* is mathematically given by: 
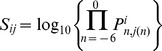
, where *j(n)* is the amino acid at position *n* in the C-terminus of peptide *j*, and 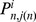 is the PWM entry corresponding to position *n* and amino acid *j(n)* for the *i* ^th^ PDZ domain. The higher the score, the more similar to the PDZ binding specificity the C-terminal sequence is. Hence, for a given threshold on PWM scores, C-terminal regions are predicted to contain a PDZ-binding motif if the PWM score is higher than the threshold value for at least one PDZ domain (Figure 1). The number of nsSNPs falling in predicted PDZ-binding C-terminal sequences was then computed for a range of threshold values on the PWM scores (Figure 1). P-values shown in Figure 1C were computed by comparing the number of nsSNPs affecting these sequences, compared to the overall frequency of C-terminal nsSNPs. For wild-type C-termini with nsSNPs affecting one of their amino acids, the PDZ binding score of each mutated sequence was computed. nsSNPs most likely to disrupt interactions are the ones affecting high scoring (>*S_max_*) C-terminal sequences and where the mutated sequence leads to a much lower score (<*S_min_*, see Figure 2). The frequency of such nsSNPs was evaluated (Figure 3, red curve) and compared to the expected frequency of random amino acid modifications (excluding synonymous ones) at the same positions. Expected frequencies and standard deviations were computed by averaging over 1000 randomizations of all nsSNPs (Figure 3, black curve).

Using the same strategy, we also analyzed all new C-termini resulting from a STOP codon truncating the wild-type protein sequence. Insertion of STOP codons is much more likely to dramatically change the protein function, and in particular to alter its C-terminal sequence. This can be either by truncating a protein with a natural PDZ-binding motif containing C-terminus, or by creating a new PDZ-binding motif containing C-terminus.

### Validation dataset

The aim of our work is to develop a computational method to identify nsSNPs most likely to disrupt or create PDZ-binding motifs. As there are currently no large experimental dataset of binding activity values between WT and naturally mutated PDZ ligands to train our model, some of the thresholds, in particular *S_min_* and *S_max_*, had to be manually estimated. To benchmark our estimates, we retrieved PDZ-mediated direct physical interactions from the manually curated PDZbase database (see Table S1) [36]. Interactions involving CASK PDZ1 were not considered since no wild-type C-terminus matches its specificity and therefore no nsSNP is predicted to disrupt its interactions (i.e., CASK does not appear in Table S2 and Table S3). 23 out of 36 interactions in PDZbase display scores higher than *S_min_*, indicating that known interactions scoring lower than *S_min_* (13 in total) are significantly underrepresented (P = 6×10^−38^, Fisher's exact test, for a universe of 296,265 interactions involving the 15 PDZ domains present both in PDZbase and in our dataset, with 293,447 scoring lower than *S_min_*).

### PDZ Domain binding analysis

**Figure 4:**
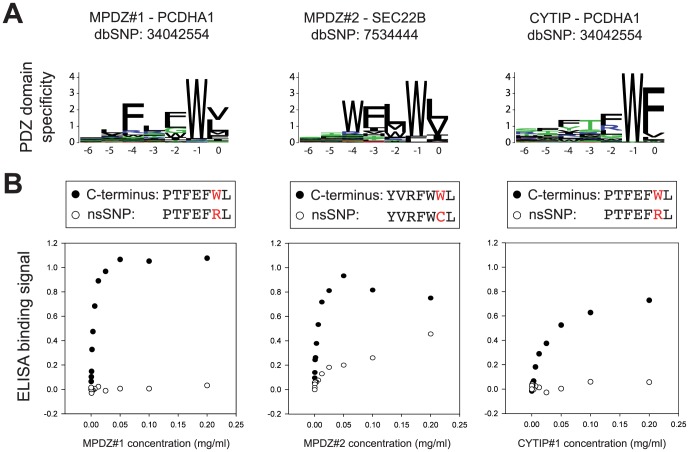
PDZ binding is affected by nsSNPs. (A) Sequence logo representing the peptide binding profile of the first and second PDZ domain of Multiple PDZ Domain Protein (MPDZ) and the PDZ domain of cytosine interacting protein (CYTIP) [19]. The interactions predicted to be disrupted by an nsSNPs are shown above. (B) Saturation binding of MPDZ#1, MPDZ#2 and CYTIP to peptides derived from sequence logos shown in Figure 4A (filled circle) and related peptides containing nsSNPs (open circles). The ELISA signal at 450 nm is plotted vs. the PDZ concentration in mg/ml. Note the C-terminus of PCDHA1 corresponds to the third isoform in UniProt (Q9Y5I3-3).

Biotinylated peptides were synthesized consisting of either the wild-type C-terminal sequence or the nsSNPs predicted to disrupt the interactions with PDZ domains. Assay plates were prepared by immobilizing the biotinylated peptide 50 μg/ml in phosphate buffered saline (PBS) on Maxisorp immunoplates coated with neutravidin (10 mg/ml, Pierce, Rockford, IL) and blocked with bovine serum albumin (0.5% w/v BSA in PBS). After 1 h incubation with GST-PDZ fusion protein at the indicated concentrations (see Figure 4), the plates were washed with PBS 0.05% Tween 20, incubated with anti-GST horseradish peroxidase antibody fusion (1∶10,000 dilution, Sigma-Aldrich, St. Louis, MO) for 30 min at 4°C. After washing with PBS 0.05% Tween, bound GST-PDZ protein was detected with TMB (3, 3′, 5, 5′ Tetramethylbenzidine) peroxide (KPL, Gaithersburg, MD). The color development was stopped with 1 M H_3_PO_4_ and the signal recorded at 450 nm in a Microplate reader (Biotek, Winooski, VT, USA).

## Results

### nsSNPs are over-represented in C-terminal sequences

nsSNPs replacing one amino acid by another one can have different effects on proteins depending on which part of the amino acid sequence they affect. For instance, nsSNPs found in protein interaction interfaces will often result in weakening or disruption of the interaction. To gain insight into nsSNPs affecting PDZ-mediated interactions, we first computed the frequency of nsSNPs in C-terminal regions, as these are the classical targets of PDZ domains. C-terminal sequences are defined here as the last seven residues of proteins, following Tonikian et al. [19]. For proteins with multiple splice variants, an nsSNP was considered as affecting the C-terminus if it was found in the last seven amino acids of any splice variant. In total 1,026,979 nsSNPs were found in all human proteins and 15,004 nsSNPs were found in C-termini. C-termini contain 151,571 amino acids out of a total of 11,269,376 in all human proteins. Therefore, the number of nsSNPs in C-terminal regions is larger than expected by chance (expected value: 13,812.7, P = 8.4×10^−26^, Binomial test), likely because protein C-termini are generally solvent-accessible and are less constrained in protein structures [18]. The same trend is observed if extracellular and mitochondrial proteins are excluded from the statistics (see Materials and Methods). In particular, 13,490 nsSNPs out of a total of 939,561 nsSNPs are observed in C-terminal regions for this filtered list of proteins. These regions consist of 138,257 amino acids out of a total of 10,424,750 for the subset of proteins considered here. As previously, the number of nsSNPs in C-terminal regions is larger than expected by chance (expected value: 12,460.8, P = 1.4×10^−21^).

### nsSNPs are slightly under-represented in PDZ-binding motifs

We then used the PWMs derived from phage display data to scan wild-type human C-termini (excluding extracellular and mitochondrial proteins) and rank them according to their score with each PDZ domain (see Methods). High scoring C-termini contain well-defined PDZ binding motifs and are predicted to interact with some PDZ domain [26]. Overall, we observe that predicted PDZ binding motifs tend in general to have slightly less nsSNPs than other C-terminal sequences for a range of thresholds over the PWM scores, roughly between -9 and -6 (Figure 1). However, significant fluctuations are observed suggesting that the trend is weak. Moreover, the highest-ranking binding motifs do not show underrepresentation of nsSNPs (P-values close to 1 in Figure 1C), suggesting that PDZ ligands can still be significantly affected by nsSNPs. Thus, predicting the effect of nsSNPs in these motifs is important to infer the possible molecular and physiological consequences of such mutations.

### Predicting nsSNPs that disrupt binding to PDZ domains

To gain insight into the effect of these mutations, we compared the similarity of both the wild type and mutated C-termini to the optimal PDZ domain specificity modeled by the different PWMs. By defining a confidence interval [*S_min_*, *S_max_*] on the PWM scores *S_ij_* (see Method), we classified nsSNPs in three categories: predicted to disrupt PDZ-binding, predicted to create a new PDZ-binding motif and predicted not to affect possible PDZ-mediated interactions (Figure 2). To fix a lower bound (*S_min_*) from which C-terminal sequences are likely not to bind to a PDZ domain, we used the results of Figure 1B and set a threshold *S_min_* = −8.3. This corresponds approximately to the point where nsSNPs start being no longer underrepresented in PDZ-motif containing C-terminal sequences. Moreover, reported interactions between PDZ domains and WT C-termini scoring lower than *S_min_* are significantly underrepresented (P = 5.8×10^−36^, see Materials and Methods).

We then explored a range of different values for *S_max_* defining PDZ-binding motifs (Figure 3). These results indicate that nsSNPs found in PDZ binding motifs and predicted to disrupt the binding are only slightly under-represented in comparison to random mutations (Figure 3). However, this is not statistically significant for large *S_max_* values. Thus, there are many nsSNPs that potentially alter PDZ-binding motifs, stressing the importance of better characterizing them at the molecular level. In Table S2, we list all instances of predicted PDZ-mediated interactions likely to be affected by nsSNPs using a threshold *S_max_* = −6.4 (i.e. interactions between a PDZ *i* and protein *j* with a PWM score *S_ij_*>*S_max_* for the wild-type C-terminus and a PWM score *S_ij′_*<*S_min_* for the mutant). In total, we identified 41 cases of PDZ motif-containing C-terminal sequence that were significantly disrupted by nsSNPs. In addition, we found 20 cases of nsSNPs falling in C-termini not predicted to bind PDZ domains, but for which the mutated sequence shows a clear PDZ-binding motif (see Table S2). This corresponds to a non-exhaustive list of the most likely candidates and we cannot exclude that some C-termini scoring slightly lower than *S_max_* may also provide true examples of nsSNPs disrupting or creating interactions.

### Experimental validation of computational predictions

To validate our computational screening, we tested three nsSNPs that we expected to alter the binding of human C-terminal peptides with PDZ domains (Figure 4). In all cases, we observed decreased binding of the PDZ domains to the ligand containing the nsSNP in comparison to the wild-type ligand. This clearly shows that nsSNPs strongly affect the *in vitro* binding to the corresponding PDZ domain. In our case, these mutations were manually selected by further inspecting the sequence logos, which considered physicochemical properties of the amino acids, information not modeled in the PWM. Therefore we cannot always expect the same success rate based purely on the PWM scores. Nevertheless, many other cases show similar patterns of clear PDZ-binding motifs that are modified by nsSNPs with large physicochemical changes (e.g. hydrophobic to positively charged residues, see Table S2).

Interestingly, the three interactions with the wild-type C-termini tested in this work correspond to proteins that have not yet been reported to interact with these PDZ domains. As such our results not only provide insights into molecular mechanisms of nsSNPs but may also suggest biologically relevant protein-protein interactions.

### Stop codons

nsSNPs resulting in a STOP codon within a coding sequence are less frequent than nsSNPs modifying one single residue, but often have dramatic effects on protein function. In particular, the C-terminal sequence will almost always be completely modified. Therefore nsSNPs resulting in STOP codons will in general strongly affect binding to PDZ domains. In total, we identified 26,864 nsSNPs that create a STOP codon before the normal C-terminal sequence of human proteins, not including extracellular and mitochondrial proteins.

Using the same threshold parameters *S_min_* and S*_max_* as above, we isolated 1,030 instances of STOP codons that either suppress (751 STOP codons affecting 225 predicted PDZ mediated interactions) or create (279 STOP codons) a C-terminal sequence predicted to bind some PDZ domain (see Table S3). Many of the STOP codons predicted to suppress PDZ-mediated interactions affect proteins known to interact with PDZ domains. For instance 19 of the 225 predicted PDZ mediated interactions are reported in BIOGRID version 3.2.104 (P = 2.5×10^−27^, Fisher's exact test, with a universe size of 533,277 consisting of all possible interactions of the 27 PDZ containing proteins present in BIOGRD and considered in this study with any of the 19,751 human proteins studied here, and 800 interactions in BIOGRID involving these PDZ containing proteins) [37].

## Discussion

Predicting the functional consequences of genetic variations is important for understanding the molecular mechanisms underlying both hereditary diseases and differences between populations. For SNPs outside of coding regions or SNPs not affecting the encoded amino acids, interpretation of functional effects is challenging, though changes in promoter or enhancer regions, DNA structure, or gene expression can sometimes explain their functional effects. Large-scale initiatives such as the ENCODE project are playing an important role to decipher these molecular mechanisms [38]. For nsSNPs, much insight can be gained by analyzing the corresponding changes at the protein sequence level. Some parts of proteins, such as core regions of protein domains, are well annotated and the molecular consequences of nsSNPs in these regions can be predicted using residue conservation or structural information. Unstructured or less-conserved regions, such as linear motifs targeted by modular protein domains, are generally less covered by existing approaches. As a result, the effect of nsSNPs falling in these regions is more difficult to predict with traditional computational methods [12].

Using a detailed computational model of PDZ domain binding specificity, we have shown that the effect of nsSNPs affecting PDZ-binding linear motifs could be accurately predicted. This problem is particularly important first because PDZ domains mediate key protein-protein interactions and second because many nsSNPs affect C-terminal sequences and some of them have already been linked to diseases [21], [22].

Our experimental validation shows that accurate predictions can be achieved for nsSNPs found in linear motifs. The experimentally observed interactions also provide interesting evidence of potentially yet undetected protein interactions. Most promising for follow-up experiments is the possible interaction between the CYTIP PDZ domain and the C-terminal peptide of PCDHA1 (isoform 3, UniProt ID Q9Y5I3-3).

Throughout this work, we have considered the last seven C-terminal amino acids as the maximum length of PDZ-binding motifs. This encompasses specificity-determining positions for most PDZ domains [19]. Some domains display non-canonical binding modes that can involve additional residues along the binding peptides [39] or internal protein sequences. However, positions outside of the main specificity determining ones are likely to play a less important role, in general for the PDZ family, and therefore nsSNPs found in them will influence less dramatically the binding. Further, we have only considered a simple filter of predicted interactions limited to removing exclusively extracellular and mitochondrial proteins. Many other factors in the cellular context contribute to determining whether a protein interaction will occur in the cell, such as co-expression, co-localization and interactor competition.

Our predictions, especially the examples listed in Table S2 and Table S3, are based on threshold values used to select nsSNPs that significantly affect PDZ binding motifs (see Figure 2). We note that these thresholds do not aim to cover all reported PDZ-binding motifs but rather to determine the ones for which the effect of nsSNPs can be predicted. For instance, PDZ domains with low specificity may have some known ligands that do not show scores higher than *S_max_*. However, in these cases, the effect of nsSNPs is more difficult to evaluate since changes at the C-terminal sequence do not result in large differences in PWM scores. Moreover, some PDZ domains display specificity profiles that do not match existing C-termini (e.g., CASK PDZ1 in our data) [40]. In these cases, determining the effect of nsSNPs is again difficult since scanning WT sequences does not result in predicted PDZ-binding motifs. These are limitations of our approach that restricts its scope and may result in missing some of the nsSNPs in specific PDZ-binding motifs. Future improvements in data available for PDZ domains and in computational prediction methods will improve this.

The presence of nsSNPs in a protein can be interpreted in multiple ways. First, for nsSNPs with high population frequency (e.g., nsSNPs on a recessive allele), the different variants may not have a major deleterious functional impact and may be well tolerated. Alternatively, nsSNPs linked to diseases or other phenotypes often have some molecular consequence such as modifying enzyme activity, destabilizing protein structures or disrupting protein interactions [41]. The methods developed in this work are especially suited for the latter case, when nsSNPs have known or potential phenotypic consequences (e.g., nsSNPs found associated with a phenotype in a genome wide associate study), for which the effects at the molecular level are to be determined. However, we also point out that our approach may be used, ideally in combinations with other existing tools [6], [8], [9], to predict which nsSNPs are more likely to have phenotypic consequences, thereby helping to prioritize further investigations [41]. This could be especially important for cancer genetics, since most mutations found in tumor cells are passengers due to high genome instability and only a few are driving the oncogenic process [42].

In this work we have focused on PDZ domains, mostly because of the availability of experimental data that enable accurate computational predictions of interactions. Yet, the strategy presented in this work could be applied to other domains such as SH3, WW or kinases. With the current increase in available genomic data coming for large population sequencing projects and cancer genomics projects, genetic variations will increasingly be uncovered. Methods such as ours, eventually expanded to consider a wider range of protein interaction domains, will help decipher the molecular mechanisms resulting from these new nsSNPs.

## Supporting Information

Table S1 **List of PDZ mediated interactions in PDZbase database involving domains considered in this work.** The PWM scores have been computed as described in Materials and Methods.
(XLS)

Table S2 **List of C-terminal sequences with nsSNPs modifying one single amino acid and predicted to disrupt or create PDZ binding motifs.**
(XLS)

Table S3 **List of C-terminal sequences with nsSNPs resulting in STOP codons and predicted to disrupt or create PDZ binding motifs.**
(XLS)
